# Recurrence and Survival Rates for 1400 Early Breast Tumors Treated with Intraoperative Radiation Therapy (IORT)

**DOI:** 10.1245/s10434-021-11295-1

**Published:** 2022-01-22

**Authors:** Melvin J. Silverstein, Melinda S. Epstein, Peter Chen, Kevin Lin, Sadia Khan, Lincoln Snyder, Colleen Coleman, Lisa Guerra, Farideh Dehkordi-Vakil, Brian Kim

**Affiliations:** 1 Department of Surgery, Hoag Memorial Hospital Presbyterian, Newport Beach, CA 92663 USA; 2 Keck School of Medicine, University of Southern California, Los Angeles, CA 90033 USA; 3 Hoag Department of Clinical Research, Hoag Memorial Hospital Presbyterian, Newport Beach, CA 92663 USA; 4 Department of Radiation Oncology, Hoag Memorial Hospital Presbyterian, Newport Beach, CA 92663 USA; 5 Center for Statistical Consulting, University of California, Irvine, Irvine, CA 92697 USA

## Abstract

**Introduction:**

Intraoperative radiotherapy (IORT) permits accurate delivery of radiation therapy directly to the tumor bed. We report local, regional, and distant recurrence data along with overall and breast cancer-specific survival for 1400 tumors treated with x-ray IORT.

**Methods:**

A total of 1367 patients with 1400 distinct tumors were enrolled in a registry trial. All received breast conservation surgery and low-energy 50 kV x-ray IORT. To be eligible for excision plus IORT as the only local treatment, histopathology had to confirm tumor size ≤30 mm, margins ≥2 mm, negative lymph nodes, and no extensive lymphovascular invasion. Patients who failed any parameters were referred for additional surgery and/or whole breast radiation therapy (WBRT).

**Results:**

There were 64 ipsilateral local recurrences, 60 were in the IORT only group, 7 axillary recurrences, and 7 distant recurrences. Forty-one local recurrences were within the same quadrant as the index cancer. Twenty-three were in different quadrants. With 62 months of median follow-up, the 5-year Kaplan–Meier probability of any event for all 1400 tumors was 5.27%. For 1175 patients who received IORT only, it was 5.98%. For favorable subtypes, it ranged from 2.41 to 4.31%. Multivariate analysis revealed that biologic subtype luminal A and the addition of WBRT significantly reduced the risk of local recurrence.

**Conclusions:**

The local, regional, and distant recurrence rates observed were comparable to those reported in the literature for IORT but higher than those reported for standard forms of WBRT, hypofractionated treatment, or APBI. IORT benefits include convenience, decreased exposure to medical environments, and low complication rates.

During the past four decades, breast conservation therapy (BCT), using a combination of surgical excision and whole breast radiation therapy (WBRT), has become the treatment of choice and the standard of care for most operable breast cancers. Equal survival for BCT compared with mastectomy was proven in multiple randomized trials.^1–4^ Recent papers have suggested that survival may now be superior for BCT.^5,6^

Unfortunately, 10–15% of women who undergo breast-conserving surgery do not complete the prescribed course of irradiation or receive no irradiation at all. Reasons cited include radiation-induced toxicities, inconvenience, cost of daily therapy, increased exposure to medical environments, commuting to and from the radiation center, and the overall logistics of WBRT.^7,8^ To improve compliance rates and to assist patients living great distances from treatment centers, alternatives to WBRT, such as accelerated partial breast irradiation (APBI), have been developed.

Intraoperative radiation therapy (IORT) delivers a single dose of radiation therapy directly to the tumor bed, during surgical excision, solving most of the above-mentioned compliance issues. IORT also can be delivered as a delayed procedure, requiring a second operation. It is less convenient and more expensive, but a second procedure permits better patient selection, as final histopathology is known.

Because 85–90% of local recurrences occur at or near the index cancer, IORT allows radiation to be delivered to the precise area where recurrence is most likely while simultaneously reducing radiation exposure to normal surrounding tissues and radiation-induced toxicity.^9,10^ Utilizing IORT for initial radiation therapy does not eliminate the potential use of WBRT due to unfavorable final tumor histopathology or for treatment of a local recurrence in the future.

Two prospective, randomized IORT trials, TARGIT-A and ELIOT, investigated the efficacy of IORT in the treatment of early breast cancer compared with standard WBRT. Recently published long-term data from these trials have shown IORT to be a safe alternative to WBRT with an low risk of local recurrence.^11–16^ While recurrence rates for IORT were higher than WBRT, ELIOT, and TARGIT-A, immediate treatment did not surpass pre-prescribed trial guidelines at 5 years, whereas TARGIT-A delivered as delayed treatment showed rates of recurrence exceeding non-inferiority criteria. The probability of local recurrence continued to increase at 10 years in both of these trials. The increased rate of local recurrence did not have a statistical impact on survival during the time period studied.

Our group began an IORT registry trial in 2010.^17,18^ We have previously reported low complication rates with this technique.^19,20^ In this report, we analyzed both recurrence and survival data for the first 1400 tumors treated with x-ray IORT at our facility.

## Methods

### Patient Population

A total of 1367 patients with 1400 distinct breast tumors (33 bilateral) and a histopathologic diagnosis of invasive ductal carcinoma, invasive lobular carcinoma, ductal carcinoma in situ (DCIS), or any combination of these diagnoses, were accrued to a prospective IORT registry trial between June 2010 and March 2020 at Hoag Memorial Hospital Presbyterian, Newport Beach, CA, USA. The trial protocol was approved by an institutional review board and met the guidelines of their responsible governmental agency. All patients were consented and provided with a copy of the written consent. The study was designed in 2010 and did not include data collection of race or ethnicity. Inclusion criteria were developed by a multidisciplinary group of radiation oncologists and breast surgeons and were modified from existing guidelines regarding accelerated partial breast irradiation (APBI) from the American Society of Breast Surgeons (ASBrS) and American Society of Therapeutic Radiation Oncology (ASTRO). Additional favorable subgroups were defined by combinations of know favorable factors, such as low-grade, older age, small size, luminal A, etc.

### Hoag Protocol Requirements

Before consent, all patients had to be at least aged 40 years and have an overall tumor extent ≤30 mm as estimated by mammography, ultrasonography and contrast-enhanced magnetic resonance imaging (MRI), unless MRI was contraindicated. Final tumor extent included all foci of cancer and was determined by the pathologist, using serial sectioning, and microscopic correlation.

All patients with invasive breast cancer were required to have negative axillary lymph nodes on frozen section. Sentinel lymph node biopsy was not required for patients with pure DCIS unless there was a preoperative suspicion of invasion.

To be eligible for IORT as the sole adjuvant radiation therapy, final histopathology had to confirm tumor extent ≤30 mm, tumor margins ≥2 mm for both invasive and noninvasive disease, no extensive lymphovascular invasion (defined as ≥3 unequivocal foci of LVI), not multifocal/multicentric, and negative axillary lymph nodes. Isolated tumor cells (N0i+) were acceptable. Patients that deviated from one or more criteria were considered protocol violations and were referred for additional surgery (reexcision or mastectomy) and/or WBRT with IORT becoming the boost. The choice of additional treatment depended on the nature of the protocol violation and was decided following a thorough discussion with the patient, her surgeon and the radiation oncologist.

If a positive lymph node was identified intraoperatively or if skin to balloon distance, as measured by intraoperative ultrasound, was <8 mm, IORT was not performed. This occurred during 66 surgeries (4.5%), 54 times due to positive lymph nodes found on frozen section, and 12 times because of inadequate skin to balloon distance. These patients are not included in this analysis, because they did not receive IORT.

### Procedure

Study participants underwent breast-conserving surgery to remove their tumor and received 20 Gy (50 kV) x-ray irradiation to the tumor bed using the Xoft Axxent Electronic Brachytherapy System^®^ (Xoft, San Jose, CA, USA, a subsidiary of iCAD, Inc.). Following IORT balloon placement, skin-to-balloon distance was measured from multiple directions with ultrasound. The minimum allowable distance for treatment was 8 mm. IORT was delivered to 1324 tumors during the initial surgical procedure and to 76 tumors as a separate delayed operation. Delayed patients generally came from outside facilities for secondary IORT, as our preference was to give IORT during the initial operation.

### Statistical Analysis

Kaplan–Meier analyses were used to estimate local recurrence and survival probabilities. The Cox Proportional Hazard model was used to examine recurrence hazard ratios for treatment methods and key characteristics, including estrogen receptor, progesterone receptor, biologic subtype, 2017 ASTRO Category, HER2-neu status, nuclear grade, age, and tumor extent. Proportionality assumption was verified through graphical methods. 95% confidence bands were calculated as described by Hall and Wellner.^21^ Analyses were performed using SAS 9.4 software (version 9.4; SAS institute Inc., Cary, NC).

## Results

Data entry was frozen July 1, 2021. In this report, we analyze the first 1400 tumors, the last of which was treated in March 2020. They had a median follow-up of 62 months and 1388 (99.2%) had been followed more than 1 year.

The characteristics of this cohort are shown in Table 1. A total of 1106 tumors (79%) were invasive (988 ductal and 118 lobular); 294 (21%) were pure DCIS. Biologic subtypes were determined for invasive cancers using immunohistochemical surrogates:^22^ 72% were luminal A; 95% were hormone receptor-positive.

**Table 1:** Characteristics of IORT trial cohort

Variable	*N* (%)
*N*	1400
Tumor type	
DCIS	294 (21%)
Infiltrating ductal	988 (71%)
Infiltrating lobular	118 (8%)
Median follow-up (range)	62 month (6 month–11 years)
Median follow-up ≥1 year	1388 (99.2%)
Median age (range)	65 years (40–95)
Median tumor span	16 mm
Hormone receptor status	
Estrogen receptor positive	1333 (95%)
Progesterone receptor positive	1186 (85%)
Immediate vs. delayed IORT	
Immediate	1324 (95%)
Delayed	76 (5%)
2017 ASTRO APBI categories	
Suitable	583 (42%)
Cautionary	511 (37%)
Unsuitable	306 (22%)
Biologic subtype (invasive only)	
Luminal A	798/1106 (72%)
Luminal B (HER2 Neg)	245/1106 (22%)
Luminal B (HER2 Pos)	31/1106 (2.8%)
HER2 Pos	3/1106 (0.3%)
Basal	29/1106 (2.6%)

All tumors were categorized using 2017 American Society for Radiation Oncology (ASTRO) APBI Criteria.^23^ Although ASTRO guidelines describe separate guidance for the use of IORT, ASTRO suitability categories helped stratify the patient population studied in this registry trial. In total, 583 tumors (42%) were categorized suitable for accelerated partial breast irradiation (APBI), 511 (37%) were cautionary, and 306 (22%) were unsuitable.

### Tumors that Met all IORT Protocol Criteria

A total of 991 tumors (71%) met all study criteria after final histopathology was determined; 984 (99.3%) of these tumors were treated with IORT as their only form of local treatment. Five of these patients elected to add WBRT to their IORT and two elected to convert to mastectomy, despite meeting all study criteria and being advised that they needed no additional local treatment.

### IORT Protocol Deviations and Treatment Following Deviations

Of note, 409 tumors (29%) deviated from one or more protocol requirements after final histopathology. There was a total of 516 deviations among these 409 tumors. Protocol deviations and treatment following deviations are summarized in Table 2. Additional treatment resulted in a total of 5 separate treatment groups (Table 3). Two of those groups (IORT alone, *n* = 1175 and IORT plus reexcision, *n* = 38) make up a cohort of partial or local breast treatment (*n* = 1213). Three of those groups (IORT plus WBRT *n* = 154, IORT plus reexcision plus WBRT, *n* = 13, and IORT followed by mastectomy, *n* = 20) make up a cohort of whole breast treatment (*n* = 187). These two cohorts are compared below.

**Table 2:** Protocol deviations and treatment

Protocol deviation groups	Treatment after protocol deviations					
	Re-excision Alone	Re-excision + WBRT	WBRT alone	Mastectomy	Refused additional treatment	Number patients
Margin	30	3	21	4	87	145
Margin, node	0	0	0	0	1	1
Margin, node, size	0	1	2	1	1	5
Margin, size	7	6	24	10	19	66
Margin, LVI	1	0	4	0	0	5
Margin, LVI, size	0	0	1	0	0	1
Margin, multifocal, node	0	1	0	0	0	1
Margin, multifocal, size	0	1	0	0	0	1
Node	0	0	25	0	4	29
Node, size	0	0	5	0	1	6
Node, LVI	0	0	5	0	0	5
Node, LVI, size	0	0	2	0	0	2
Size	0	0	54	2	48	104
Size, multifocal	0	0	2	0	1	3
Multifocal	0	1	0	0	1	2
LVI	0	0	4	1	28	33
Total	38	13	149	18	191	409

409 patients experienced 1 or more protocol violations. They redivided into 16 groups

**Table 3:** Breakdown of treatment and local recurrences

Treatment	*N*	# Local recurrences	5-year probability (%)
IORT alone	1175	60	5.98
IORT + WBRT	154	1	0.68
IORT + reexcision + WBRT	13	0	0.0
IORT + reexcision	38	2	9.09
IORT + mastectomy	20	1	0.0
Total	1400	64	5.27

Almost half the patients with protocol deviations, 191 of 409 (47%), declined any additional local treatment, bringing the total number of tumors treated with IORT alone to 1175 (191 with protocol deviations plus 984 who met all criteria). Of the 218 remaining patients (409-191) with protocol deviations, 149 received WBRT, 13 underwent reexcision followed by WBRT, 38 underwent reexcision alone, and 18 converted to mastectomy. The 5 treatment groups, the number of local recurrences in each group, and the 5-year probability of local recurrence for each group are summarized in Table 3.

### Recurrences and Survival

There were 64 local recurrences: 47 were invasive; 17 were pure DCIS; 41 of 64 (64%) local recurrences were in the same quadrant as the index cancer; 23 local recurrences were in quadrants different from the index cancer; 94% of local recurrences (60 of 64) were in the IORT only treatment group. The median time to local recurrence was 38 (range 7–117) months. In addition to 64 local recurrences, there were 7 axillary recurrences and 7 distant recurrences. These 78 events occurred among 69 patients. One patient died of metastatic breast cancer, whereas 44 others died of nonrelated causes.

Table 4 summarizes the probability of local recurrence for various cohorts of patients. The 5-year probability of any event in any quadrant of the breast for all 1400 tumors was 5.27%. For 1175 patients who received IORT as their only form of local treatment, it was 5.98%. A section entitled Favorable Subgroups is included in Table 4. It lists four selected subgroups with lower 5-year probabilities of local recurrence: for 583 patients who met the requirements to be designated as ASTRO suitable, it was 4.31%; for Nottingham Grade 1 invasive carcinomas, it was 2.91%; and for patients ≥70 years, with luminal A tumors, 20 mm or less, it was 2.41%.

**Table 4:** Kaplan–Meier calculated 5-year probability of local or distant recurrence or survival for various study subgroups

Recurrence location and type	*N*	No. events	5-year probability (%)
All local recurrences (DCIS + Inv) All quadrants	1400	64	5.27
All local recurrences (DCIS + Inv) Same quadrant	1400	41	3.26
Invasive local recurrences All quadrants	1400	47	3.83
Invasive local recurrences Same Quadrant	1400	31	2.43
Favorable categories			
ASTRO suitable	583	18	4.31
Nottingham Grade 1	452	9	2.91
Age ≥70, Luminal A, Span ≤2 cm	207	4	2.41
Luminal A or B and Span ≤10 mm	268	9	3.73
All local recurrences by whole breast or partial breast treatment			
Whole breast treatment (Received WBRT or mastectomy in addition to IORT)	187	2	0.2
Partial breast treatment (Received IORT alone or IORT plus reexcision)	1213	62	6.07
Pure DCIS patients			
DCIS Tumors (All recurrences)	294	16	6.76
DCIS Tumors (Inv recurrences)	294	8	3.26
By local treatment			
IORT only (All recurrences)	1175	60	5.98
IORT plus WBRT only	154	1	0.68
IORT only (Local Inv Rec)	1175	44	4.34
IORT only (Same Quad Inv Rec)	1175	28	2.65
IORT no WBRT all patients	1233	63	5.93
IORT plus WBRT all patients (includes 13 re-ex + WBRT patients)	167	1	0.63

Axillary and distant recurrences	*N*	Events	5-year probability
Axillary recurrences	1400	7	0.34
Distant recurrences	1400	7	0.50

Survival	*N*	Deaths	5-year probability
Breast cancer-specific survival	1400	1	99.9
Overall survival	1400	45	96.3

For 191 patients who failed one or more IORT protocol requirements but refused additional treatment, the 5-year local recurrence probability was 8.0%. However, for 167 patients who failed one or more protocol requirement and accepted additional WBRT, it was 0.68%.

An invasive event in the same quadrant of the breast as the index cancer had a 5-year probability of 2.43%. The 5-year probability of axillary recurrence was 0.34% and of a distant recurrence, 0.50%. Overall survival at 5-years was 96.3% and breast cancer specific survival was 99.9%.

### Multivariate Analysis

We evaluated nine factors thought to be possible contributors to the risk of local recurrence (Table 5). These included five local treatment groups (Table 3), estrogen and progesterone receptors, biologic subtype, 2017 ASTRO Category, HER2-neu status, nuclear grade, age, and tumor extent.

**Table 5:** Multivariate analysis of factors related to local recurrence

	No. patients	Cox univariate			Cox multivariate		
		Hazard ratio	95% CI	Wald *p* value	Hazard ratio	95% CI	Wald *p* value
Final treatment	1400						
Local treatment^a^	1213	5.53	1.73, 33.74	0.017	8.43	2.17, 55.47	0.0062
Whole breast Rx^b^	187	1					
Estrogen receptor	1400						
Positive	1333	0.270	0.144, 0.563	0.0001	1.113	0.376, 3.75	0.85
Negative	67	1			1		
Progesterone receptor	1400						
Positive	1186	0.523	0.309, 0.925	0.0198	0.915	0.448, 2.03	0.82
Negative	214	1			1		
Biologic subtype (invasive only)	1106						
Luminal A	798	0.371	0.21, 0.654	0.0006	0.488	0.246, 0.994	0.043
Not luminal A	308	1			1		
2017 ASTRO category	1400						
Suitable	583	0.585	0.330, 0.993	0.055	0.841	0.404, 1.73	0.64
Cautionary or unsuitable	817	1			1		
Her2-neu							
(invasive only)	1106						
Negative	1071	1			1		
Positive	35	4.02	1.65, 8.43	0.0007	2.046	0.776, 4.86	0.12
Nuclear grade	1400						
High (3)	185	2.53	1.45, 4.27	0.0007	1.41	0.62, 3.06	0.39
Low (1 + 2)	1215	1			1		
Age (year)	1400						
<60	448	1			1		
61–70	452	0.754	0.410, 1.36	0.35	0.723	0.339, 1.51	0.39
71–80	416	0.723	0.372, 1.36	0.32	0.674	0.32, 1.41	0.30
>80	84	1.19	0.401, 2.87	0.72	1.155	0.37, 3.00	0.84
Tumor extent (size)	1400						
	1400	1.004	0.985,1.02	0.66	1.016	0.983, 1.04	0.78

^a^Local treatment includes excision plus IORT (*n* = 1175) or excision plus reexcision plus IORT (*n* = 38; *N* = 1213)

^b^Whole-breast treatment includes excision plus IORT plus WBRT (*n* = 154), or excision plus IORT plus reexcision plus WBRT (*n* = 13), or excision plus IORT followed by mastectomy (*n* = 20; *N* = 187)

We initially examined the recurrence hazard using all five different treatment groups. In two of those groups (IORT + mastectomy and IORT + re-excision + WBRT), there were no recurrences, and the Firth correction was used to get parameter estimates and the 95% confidence bond.

Next, we simplified the analysis by creating two treatment groups as described above: 187 who received whole breast treatment (154 IORT plus WBRT, 13 IORT plus reexcision plus WBRT, and 20 who received IORT followed by mastectomy) versus 1213 who received localized treatment (1175 IORT only and 38 IORT plus reexcision).

Using Cox proportional hazard regression model, Table 5 shows that by univariate analysis, whole breast treatment, estrogen and progesterone receptor positivity, Luminal A subtype, Astro category suitable, HER2-negativity, and low nuclear grade were all significant predictors of a lower local recurrence rate.

By multivariate analysis, after adjustment for the confounding variables listed in the table, only two factors remained significant. Patients in the localized breast treatment group had a significantly higher local recurrence (almost 9 times) than those in the whole breast treatment group (hazard ratio, 8.43; 95% confidence interval [CI], 2.17–55.47; *p* 0.006). Patients with biological subtype luminal A have 51.2% lower hazard of local recurrence compared with patients that are not luminal A (hazard ratio, 0.488; 95% CI, 0.246–0.994; *p* 0.043), and patients who are HER2-positive have two times the hazard of local recurrence compared with patients that are HER2-negative, but this increase in hazard is not statistically significant at 5% level (hazard ratio, 2.046; 95% CI, 0.776–4.86; *p* 0.12).

**Fig. 1:**
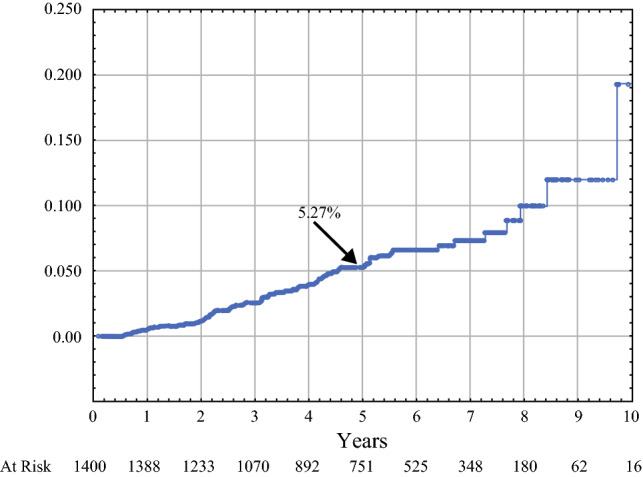
Kaplan–Meier analysis of the probability of local recurrence. The local recurrence probability at 5 years is projected to be 5.27%. This includes all ipsilateral breast tumor events, including those in quadrants other than the index cancer

**Fig. 2:**
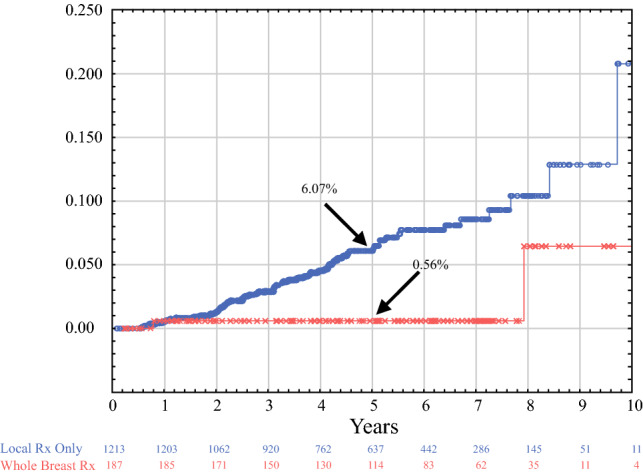
Cases are divided into two groups: 187 who received whole-breast treatment (red), and 1213 who received treatment to the local area only (blue). The 5-year probability of recurrence are 0.56% and 6.07%, respectively (*p* < 0.005)

Figure 1 shows the Kaplan–Meier probability of local recurrence in all 1400 tumors. Figure 2 shows the recurrence probability when all 1400 tumors are divided into two group, as was done in the multivariate analysis (Table 5): 187 with whole breast treatment versus 1213 with localized breast treatment to the site of the index cancer.

## Discussion

The local management of breast cancer has trended toward deescalation in recent years: sentinel node excision rather than axillary dissection, omission of sentinel node biopsy for selected favorable older patients, accelerated courses of WBRT, and the development of APBI. IORT continues the trend toward less treatment.

Randomized trials, comparing mastectomy with excision plus WBRT, yielded equivalent survival and set the standard for the last 40 years.^1–4,11,16^ Now, two randomized IORT trials have shown that survival is equivalent when excision plus WBRT is compared with excision plus IORT, but with an increased risk of local recurrence for IORT.^11,16^

IORT has been available in the United States since the mid-2000s, but its use has been minimal. The initial problem was a lack of long-term efficacy data. Those data are now available with the publication of TARGIT A and ELIOT updates, 20 years after they were initiated. Both trials have produced low local recurrence rates without a negative impact on survival.^11,16^

The ELIOT Trial used electrons to deliver 21 Gy in a single dose to the tumor bed versus WBRT with conventional fractionation (50 Gy given as 25 fractions of 2 Gy, plus a 10 Gy boost).^11^ With a median follow-up of 12.4 years, the 5-, 10-, and 15-year local recurrence rates for IORT were 4.2%, 8.1%, and 12.6% compared to 0.5%, 1.1%, and 2.4% for patients treated with WBRT (*p* < 0.0001). Despite an approximately fivefold difference in local recurrence rate for IORT and a significant difference in axillary recurrences (1.9% for IORT vs. 0.3% for WBRT, *p* = 0.01), there was no significant difference in the rate of distant disease, overall survival, or breast cancer-specific survival. The ELIOT Trial Group concluded that IORT should be offered to selected patients at low risk of local recurrence. ELIOT identified a particularly low-risk group as tumor ≤1 cm, well-differentiated, luminal A, with a Ki67 < 14%. Of note, only 5% of ELIOT IORT patients (those with ≥4 positive nodes) received supplemental WBRT.^11^

TARGIT A used photons (50 kV x-ray) to deliver 20 Gy at the surface of an applicator. The patients were randomized so that half received conventional WBRT to a dose of 50 Gy with or without a tumor bed boost. The experimental IORT arm was “risk-adapted,” meaning that if final pathology showed certain prespecified high-risk factors, WBRT was added, and the previously given IORT became the boost.^12,14,16^

TARGIT A reported their long-term follow-up as two distinct patient subgroups: immediate (those who received IORT during their initial surgery) and delayed (those who received IORT as a secondary procedure). The immediate group consisted of 2298 patients with a median follow-up of 8.6 years. All patients in the IORT arm received IORT during their original surgery; 28.6% received additional WBRT. Unfortunately, the results of patients receiving IORT only were not presented separately. The Kaplan–Meier estimate of probability of local recurrence at 5 years was 2.23% for IORT and 1.02% for WBRT (*p* = 0.28).^16^

The second TARGIT A subgroup consisted of 1153 patients with a median follow-up of 9 years.^13^ All patients in the IORT arm received IORT as a delayed procedure, requiring a second operation. The probability of local recurrence at

5 years was 3.96% for IORT and 1.05% for WBRT (*p* = 0.052), exceeding the 2.5% limit for noninferiority. It is surprising that the delayed TARGIT A group had a higher local recurrence probability than the immediate TARGIT A group, because the final pathology was already known and favorable. This disparity can likely be explained by the fact that only 5.8% of the delayed group received supplemental WBRT, whereas 26.8% of the immediate group, using the “risk adapted” approach received supplemental WBRT.

TARGIT R, a nonrandomized IORT study that included 667 patients from 12 institutions with a median follow-up of 5.1 years,^24,25^ reported a 5-year local recurrence probability of 8.0% for those who received IORT alone versus 1.7% for those who received IORT plus WBRT; 25.5% of TARGIT R patients received supplemental WBRT. When both groups were combined, the probability of local recurrence for all patients was 6.6%.

Our trial consisted of 1400 tumors treated at a single facility, by the same group of surgeons and radiation oncologists, during a 10-year period. The probability of local recurrence at 5 years was 5.27% for all patients (1400 tumors). This included all events in all quadrants (Fig. 1). Although IORT resulted in a higher local recurrence risk over current standard treatments and may be considered unacceptable by some, the absolute increase in local recurrence must be weighed against patient convenience, compliance, decreased toxicities and costs. Even compared with 5-fraction APBI and WBI data, IORT still has convenience and compliance advantages. The argument is similar to the discussion of avoiding whole breast irradiation in elderly patients based on the CALGB results, despite a fivefold increase in local recurrence.^26^

Our group also used a “risk-adapted” approach like TARGIT A to select patients for supplemental WBRT. In our study, patients who violated any of the entry criteria stated in the Methods section above were directed to receive additional local treatment. However, 191 of 409 (47%) patients who violated one or more criteria declined any additional local treatment. Of the 191 patients who refused additional treatment, 13 experienced a local recurrence with a 5-year probability of 8.0%, whereas 167 patients with protocol violations who accepted supplemental WBRT experienced a local recurrence rate of only 0.63%. This demonstrates the importance of adding WBRT to IORT if high-risk features are encountered.

To better understand IORT as the only form of adjuvant local treatment in addition to surgery, we analyzed a subgroup of 1175 patients who received IORT alone without reexcision, the addition of WBRT, or conversion to mastectomy. They experienced 60 local recurrences and their 5-year probability of a local recurrence was 5.98%. This is a fairly high rate of local recurrence but lower than that seen in TARGIT R. The high rate of local recurrence can be explained by the fact that 190 of these patients failed one of more protocol criteria and should have received additional local treatment but refused.

As shown in Table 3, the risk of local recurrence is higher for patients that received IORT only. The higher recurrence risk can be countered to some extent by better patient selection. Table 4 has a section entitled Favorable Categories. The 5-year probability of local recurrence for ASTRO suitable was 4.31%, for Nottingham Grade 1 tumors, it was 2.91%, and for patients aged ≥70 years with ≤2-cm luminal A tumors, it was 2.41%. These data support stricter selection criteria for IORT.

Supplement WBRT plays a profound role in IORT patients. Those who received it, in our trial, had a recurrence rate lass than 1.0%. When IORT is not being studied as a boost, supplemental WBRT is only offered to patients with poor prognostic findings on final histopathology. These are the patients most likely to experience local recurrence based on final histopathology. Despite this, the rates of local recurrence for those who received supplemental WBRT were significantly lower in our series and most reported trials. Since the percentage of patients receiving supplement WBRT varies from trial to trial, the overall local recurrence probabilities between trials cannot easily be compared. Of our patients, 11.9% received supplemental WBRT compared with 26.8% for TARGIT A (immediate treatment group), 25.5% for TARGIT R, and 5% for ELIOT.^12–14,25^

Because most trials report patients using intention-to-treat rules, the greater the number of patients who receive supplemental WBRT in a given cohort, the lower the overall local recurrence rate will be. The purest way to analyze IORT data might be to report all patients who met institutional requirements and were treated with IORT only. At our facility, this is a cohort of 984 patients with a 5-year probability of local recurrence of 5.57%. Given this local recurrence rate, we are continuing to rethink and revise our selection criteria for IORT patients.

The results of our multivariate analysis confirm the effect of WBRT on reducing the local recurrence rate. The result is not surprising. WBRT treats the entire breast and lower axilla. IORT irradiates approximately one centimeter around the excision cavity. One cannot expect IORT to perform at the same level as WBRT. It cannot control the development or progression of new or previously existing cancers in other quadrants. It also cannot be expected to control cancers in the same quadrant that are more than one centimeter from the applicator surface.

Multivariate analysis revealed that HER2-neu positivity to be associated with a twofold (not significant *p* = 0.12) increase in local recurrence hazard ratio. The lack of significance is likely due to the small number of HER2-positive patients There were 35 HER2-positive tumors in this series (32 luminal B HER2-positive and 3 hormone receptor-negative, HER2-positive). They experienced seven local recurrences (20%). None were treated with neoadjuvant trastuzumab-based chemotherapy, because our protocol does not allow neoadjuvant chemotherapy or hormonal therapy. Going forward, we will likely not offer IORT to HER2-positive patients.

IORT greatly simplifies the delivery of post-excisional breast irradiation. For many, it is eliminated, allowing patients to move quickly from local treatment into the systemic phase of therapy if required. When used as the only adjuvant breast irradiation, IORT eliminates anywhere from 5 to 30 outpatient visits, depending on the course of radiation therapy chosen. IORT makes breast conservation possible for women who cannot be available for 1-6 weeks of conventional, hypofractionated, or ultra-hypofractionated radiation therapy. In our patient cohort, between 10,000 and 100,000 patient-hours were saved, depending on the radiotherapy schedule chosen. Furthermore, IORT reduces a patient’s exposure to hospital and/or cancer center environments, which was of great importance, during the COVID-19 pandemic.

IORT data published to date has shown a higher probability of local recurrence when IORT alone is compared with WBRT.^11,16^ The hazard ratio increases anywhere from twofold to tenfold. The EBCTCG meta-analysis^27^ demonstrated that one breast cancer death was added by year 15 for every four local recurrences at year 10. The local recurrence increases after IORT had no significant impact on survival even in the ELIOT Trial with 12.4 years of median follow-up.^11^ This difference may be due to the closer follow-up, early detection in IORT patients, the lower-risk category of most IORT patients, and better treatment of metastatic disease when it occurs. If there are survival differences, 15–20-year data may be needed to show them.

IORT came of age in an era when hypofractionated whole breast radiation was not widely available. Compared with standard conventional WBRT, IORT greatly simplified the delivery of post-excisional breast irradiation, often eliminating 25–35 outpatient visits, and making adjuvant radiation possible for women who could not comply with a conventional course.

In the current era, the standard of care is shifting toward hypofractionation (15–16 treatments) or even ultra-hypofractionation (5 treatments), based on published data showing the equivalence of these techniques in terms of local control.^28–30^ Within this spectrum, single-fraction IORT remains the most convenient form of accelerated treatment, albeit at the price of a higher local recurrence rate. If an enlarging percentage of IORT patients continue to require the addition of WBRT and if there are randomized long term-data showing equivalence of ultra-hypofractionation with WBRT, the convenience of IORT may no longer justify its continued use. However, at the current time, improved patient selection may be able to reduce recurrences as well as the need for adding WBRT, maximizing the convenience for a carefully selected population.

For each individual patient and physician, the question will be: Are you willing to accept a significant increased risk of local recurrence, which does not appear to impact survival, in exchange for a single precise dose of radiation that will save time, costs, exposure to medical environments, and the risk of damage to surrounding and underlying tissue?

Local recurrence can be devastating both emotionally and financially. It may lead to feelings of failure on the part of both patient and physicians, and it will require additional treatment, decreasing the financial benefit of single dose treatment. We have treated most local recurrences, following IORT, conservatively with excision and WBRT, although there are no long-term data supporting this approach.

## Conclusions

The local, regional, and distant recurrence rates for IORT observed in this trial were comparable to those reported in the prospective randomized TARGIT-A and ELIOT Trials that compared IORT to WBRT. Our overall local recurrence rate was significantly higher in patients who received IORT alone compared with those who received IORT plus WBRT, but it is clearly possible to lower the local recurrence rate by selectively treating only lower-risk patients, such as ASTRO suitable or Nottingham Grade 1 or patients ≥70 years with luminal A, T1 tumors. Given the recurrence risks, even with more favorable patients, improvements are still needed in the IORT patient selection process. Regardless of what selection process we use, the rate of local recurrence for IORT will always be higher than that of WBRT. The benefits of choosing IORT include decreased treatment time, decreased exposure to medical environments, low complication rates, and lower cost if recurrences can be avoided. Patients, with the help of their physicians, will have to decide whether the benefits outweigh the risks in their particular case and those of us who provide IORT will have to continue to refine the patient selection process.
